# Trafficking Dynamics of PCSK9-Induced LDLR Degradation: Focus on Human PCSK9 Mutations and C-Terminal Domain

**DOI:** 10.1371/journal.pone.0157230

**Published:** 2016-06-09

**Authors:** Steve Poirier, Hocine Ait Hamouda, Louis Villeneuve, Annie Demers, Gaétan Mayer

**Affiliations:** 1 Laboratory of Molecular Cell Biology, Montreal Heart Institute Research Center, QC, Canada; 2 Département de Pharmacologie, Faculté de Médecine, Université de Montréal, QC, Canada; 3 Faculté de Pharmacie, Université de Montréal, QC, Canada; Tohoku University, JAPAN

## Abstract

PCSK9 is a secreted ligand and negative post-translational regulator of low-density lipoprotein receptor (LDLR) in hepatocytes. Gain-of-function (GOF) or loss-of-function (LOF) mutations in *PCSK9* are directly correlated with high or low plasma LDL-cholesterol levels, respectively. Therefore, PCSK9 is a prevailing lipid-lowering target to prevent coronary heart diseases and stroke. Herein, we fused monomeric fluorescent proteins to PCSK9 and LDLR to visualize their intra- and extracellular trafficking dynamics by live confocal microscopy. Fluorescence recovery after photobleaching (FRAP) showed that PCSK9 LOF R46L mutant and GOF mutations S127R and D129G, but not the LDLR high-affinity mutant D374Y, significantly accelerate PCSK9 exit from the endoplasmic reticulum (ER). Quantitative analysis of inverse FRAP revealed that only R46L presented a much slower trafficking from the *trans*-Golgi network (TGN) to the plasma membrane and a lower mobile fraction likely suggesting accumulation or delayed exit at the TGN as an underlying mechanism. While not primarily involved in LDLR binding, PCSK9 C-terminal domain (CTD) was found to be essential to induce LDLR degradation both upon its overexpression in cells or *via* the extracellular pathway. Our data revealed that PCSK9 CTD is required for the localization of PCSK9 at the TGN and increases its LDLR-mediated endocytosis. Interestingly, intracellular lysosomal targeting of PCSK9-ΔCTD was able to rescue its capacity to induce LDLR degradation emphasizing a role of the CTD in the sorting of PCSK9-LDLR complex towards late endocytic compartments. Finally, we validated our dual fluorescence system as a cell based-assay by preventing PCSK9 internalization using a PCSK9-LDLR blocking antibody, which may be expended to identify protein, peptide or small molecule inhibitors of PCSK9.

## Introduction

Subendothelial retention of low-density lipoproteins (LDL) in the arteries is a key initiating event in atherogenesis often leading to coronary heart diseases (CHD) or stroke [1]. Familial hypercholesterolemia (FH) is a common genetic disorder associated mostly with mutations at *LDLR*, *APOB* and *PCSK9* loci, clinically characterized by high levels of circulating LDL particles and premature CHD [2]. Proprotein convertase subtilisin-kexin type 9 (*PCSK9*; [3]) was shown to be a natural inducer of LDLR degradation [4], thereby rising LDL particle levels in the bloodstream. While PCSK9 gain-of-function (GOF) mutations can result in high plasma levels of LDL [5], PCSK9 loss-of-function (LOF) mutations lead to life-long reduction of circulating LDL and can significantly reduce major CHD events in humans [6, 7]. Therefore, it is awaited that pharmacological inhibition of PCSK9 will also result in protection against CHD [7].

In the adulthood, *PCSK9* is highly expressed in liver and to a lesser extent in other cholesterogenic tissues such as the intestine and kidneys [3] and is positively regulated by statins (HMG-CoA reductase inhibitors; [8]) through sterol regulatory element-binding protein (SREBP)-2 [9] cooperatively with hepatocyte nuclear factor (HNF)-1 alpha [10]. *PCSK9* encodes for a secreted 692-amino acid (aa) glycoprotein structurally composed of a signal peptide (aa 1–30), prosegment (pro; aa 31–152), catalytic (Cat; aa 153–454) and C-terminal cysteine-histidine-rich (CTD; aa 455–692) domains [11]. Within the endoplasmic reticulum (ER), the zymogen proPCSK9 is synthesized as a ~74 kDa protein that undergoes autocatalytic intramolecular cleavage at position 152 to form a ~14 kDa inhibitory prosegment that remains noncovalently bonded to the ~60 kDa mature PCSK9 [3, 12, 13]. This tightly bound heterodimeric complex forming an inactive enzyme is absolutely required for ER exit and secretion. An elegant study revealed that transport of PCSK9 from the ER to the Golgi apparatus requires the SEC24A subunit to be incorporated into coat protein complex II (COPII)-coated vesicles [14]. In addition, our recent work demonstrated that, independently of its chaperone activity, GRP94 binds PCSK9 in the ER and prevents premature LDLR degradation [15]. Although their *in vivo* roles on PCSK9 function are questionable [16], sortilin [17] and amyloid precursor-like protein 2 [18] were identified as sorting receptors assisting PCSK9 secretion and trafficking towards late endocytic compartments, respectively. A body of evidence indicates that PCSK9 targets LDLR for degradation by two pathways: an intracellular one from the *trans*-Golgi network (TGN) directly to late endosomes/lysosomes, involving clathrin light chains [19], and an extracellular one [20, 21] requiring the cytosolic adaptor ARH (autosomal recessive hypercholesterolemia) and mediated by clathrin heavy chain endocytosis of cell surface PCSK9-LDLR complex [22, 23]. However, the relative contributions of those molecular trafficking components in each of the cellular pathways remain to be determined.

Biochemical data showed that the surface of PCSK9 catalytic domain, together with its N-terminus released after prosegment cleavage, directly interacts with the first extracellular epidermal growth factor-like repeat (EGF-A) of LDLR [24, 25], and that the affinity of this interaction greatly increases at acidic pH [11, 26]. PCSK9-D374Y GOF mutant has a markedly increased affinity for LDLR by allowing a hydrogen bond to form at neutral pH with H306 of the LDLR EGF-A domain [25–27], resulting in an extremely severe FH phenotype [28]. Interestingly, removal of the N-terminal acid stretch (aa 31–53) of PCSK9 prosegment also strongly increases its binding to LDLR [25, 29]. In addition, it was shown that PCSK9 residue L108 makes van der Waals interactions with the LDLR β-propeller domain, which could be potentiated in L108R, S127R or D129G prosegment PCSK9 GOF mutations [30, 31]. Patients harboring the PCSK9 prosegment R46L LOF mutation had ~50% reduction in incidence of coronary events owing to a lifelong reduction in LDL-cholesterol of only 15% [6]. It has been found that the R46L variant does not affect PCSK9 endocytosis but has a 2-fold weaker affinity for LDLR resulting in a slight reduction of its capacity to induce LDLR degradation [26]. Loss- or gain-of-function mutations in PCSK9 prosegment (e.g. R46L, S127R, D129G) or CTD (e.g., R496W and H553R), which are not primarily involved in LDLR EGF-A binding, show that those domains also participate in the regulation of PCSK9-induced LDLR degradation by a mechanism for which we possess very little information.

Independently of its catalytic activity [32], PCSK9 binds to LDLR and prevents its cell surface recycling by rerouting the receptor to late endocytic compartments for degradation (reviewed in [23]). So far, two prevailing mechanisms have been proposed to explain the PCSK9-induced LDLR degradation both relying on the essential role of the PCSK9 CTD in the process [33]. Firstly, once in acidic compartments, the affinity of PCSK9 catalytic domain for LDLR EGF-A is greatly increased and could allow interaction of the CTD with the LDLR ligand-binding domain [34], creating a conformational change in LDLR that would induce its shedding by γ-secretase and its degradation in lysosomes [35]. Secondly, it has been proposed that a putative transmembrane protein would connect PCSK9 *via* its CTD to cytosolic adaptors in order to target the PCSK9-LDLR complex to lysosomes [36]. Although the exact role of PCSK9 CTD requires more investigations, it has been shown that Annexin A2 [37, 38] or a monoclonal antibody [39] that specifically bind to the CTD both inhibited the PCSK9-induced LDLR degradation.

In the present study, we developed a dual fluorescence cell-based assay and analyzed the trafficking dynamics of PCSK9 and LDLR both for intra- and extracellular pathways by live confocal microscopy. Our data revealed that PCSK9 CTD increases LDLR-mediated PCSK9 endocytosis and PCSK9 subcellular localization at the TGN. Moreover, fusion of the transmembrane domain and cytosolic tail of the lysosome-associated membrane protein-1 (Lamp1) to PCSK9 lacking the CTD (PCSK9-ΔCTD) fully restored its capacity to induce LDLR degradation, suggesting a central role of the CTD as a trafficking determinant for the PCSK9-LDLR complex. Comparative fluorescence recovery after photobleaching (FRAP) analyses showed that the LOF R46L mutation in PCSK9 is associated with higher retention at the TGN. Using a PCSK9-LDLR blocking monoclonal antibody, we validated our cell-based assay that could be used to screen for functional knockdown libraries, biologics or small molecule inhibitors.

## Materials and Methods

### Reagents and plasmids

Human neutralizing recombinant anti-PCSK9 antibody (IgG1) was obtained from BPS Bioscience (Cat. #71207, lot #121204-D). TO-PRO-3 iodide was obtained from Life Technologies (Cat. #T3605). Recombinant human PCSK9 (rhPCSK9; aa 31–692) and cDNAs encoding for full-length wild-type (WT) human PCSK9 and its GOF mutants D374Y and F379A, with a C-terminal V5 tag, cloned into pIRES2-EGFP were produced as described previously [40]. Subcloned pIRES2-EGFP plasmids encoding V5-tagged PCSK9 CTD (aa 1-31(Q31N)-405-692) and PCSK9-ΔCTD (aa 1–454), Timp1- and PCSK9-V5-TM-Ct-Lamp1 chimeras (WT, CTD or ΔCTD) and WT V5-tagged human LDLR were generous gifts from Dr. Nabil Seidah (Institut de Recherches Cliniques de Montréal). PCSK9-F379A-V5-TM-Ct-Lamp1 was generated by subcloning the F379A cassette from pIRES-PCSK9-F379A-V5 into pCMV3-hPCSK9-V5-TM-Ct-Lamp1 vector. Other PCSK9 and LDLR mutants were generated by QuickChange II XL site-directed mutagenesis (Agilent, Cat. #200521) in the pIRES-hPCSK9-V5 and pIRES-hLDLR-V5 cDNA backbones, respectively. PCSK9-Δhinge-CTD (aa 1-31(Q31N)-440-692) was generated by two-step overlap PCR using the pIRES-hPCSK9-CTD-V5 as template with the following oligonucleotides: PCR1; 5’-GGGCGGTAGGCGTGTACGGTGG, 5’-GGCCACCAGTTTGGCAGAGAAGTGGATCAG and PCR2; 5’-CTCTGCCAAACTGGTGGCCGCCC, 5’-CGCACACCGGCCTTATTCCAAG and subcloned into the pIRES-V5 vector. The monomeric fluorescent Cherry coding cDNA was fused to PCSK9 C-terminus using pCMV-Cav1-mCherry as a template (Cat. #27705, Addgene) as described previously [40]. As indicated, all PCSK9 mutants were subcloned from corresponding pIRES-hPCSK9-V5 into the pCMV-hPCSK9-mCherry backbone cDNA. Enhanced green fluorescence protein (EGFP) was fused to LDLR cDNA at its C-terminal (pCMV-hLDLR-EGFP) by PCR amplification and subcloned at AgeI/NotI into pIRES-hLDLR-V5 resulting in deletion of the V5-tag sequence and the internal ribosomal entry site (IRES). All selected clones were verified by DNA sequencing.

### Cell culture and transfections

Human hepatoma HepG2 cells were routinely cultivated in Dulbecco’s modified Eagle’s medium (DMEM; Cat. #319-005-CL, Wisent) supplemented with 10% Fetal Bovine Serum (FBS; Cat. #080–350, Wisent). Human embryonic kidney 293 (HEK293) cells were cultivated in complete DMEM without sodium pyruvate (Cat. # 319-015-CL, Wisent). HepG2 were transfected with Lipofectamine 3000 (Cat. #L3000008, Life Technologies) or XtremeGENE 9 (Cat. #06365787001, Roche Diagnostics) according to the manufacturer’s recommendations. HEK293 cells were transfected with linear polyethylenimine MW 25,000 (PEI; Cat. #23966, Polysciences) at ratio of 0.8:0.2 PEI (μg):DNA (μg) per cm^2^ of cell surface area.

### Western blot analyses

Cells were washed three times in phosphate-buffered saline (PBS) and lysed in 1x RIPA buffer (50 mM Tris/HCl, pH 8.0, 1% (v/v) Nonidet P40, 0.5% sodium deoxycholate, 150 mM NaCl and 0.1% (v/v) SDS) supplemented with 1x Complete Protease Inhibitor Mixture (Cat. #11 697 498 001, Roche Applied Science). Proteins from cell lysates and conditioned media were resolved by 8% Tris-Glycine SDS-PAGE and blotted on HyBond nitrocellulose membranes (Cat. #162–0115, Bio-Rad), blocked for 1 h in Tris-Buffered Saline-Tween 20 (TBS-T; 50mM Tris-HCl, pH 7.5, 150 mM NaCl, 0.1% Tween 20) containing 5% non-fat dry milk. Membranes were then incubated overnight in TBS-T supplemented with 1% non-fat milk and indicated antibodies: rabbit anti-PCSK9 [41] (1:2500), goat anti-human LDLR (1:1000; Cat. #AF2148, R&D Systems), mouse anti-V5-tag (1:5000; Cat. #A00641, GenScript), rabbit anti-β-actin (1:5000; Cat. #A2066, Sigma-Aldrich). Appropriate HRP-conjugated secondary antibodies (1:10,000, GE healthcare) were used for detection using the Western Lightning Ultra chemiluminescence kit (Cat. #NEl112001EA, PerkinElmer) and BioFlex EC Films (Cat. #CLEC810, InterScience).

### Immunocytochemistry and live-cell imaging

Cells plated on glass-bottom culture dishes (Cat. #P35G-0-10-C, MatTek) were washed three times with PBS and fixed with 4% paraformaldehyde for 15 min. Following extensive PBS washes, cells were permeabilized or not, as indicated in figure legends, with 0.1% Triton X-100/PBS for 10 min and incubated with 150 mM glycine to stabilize the aldehydes. The cells were then incubated for 30 min with 1% BSA (Fraction V; Cat. #BP1605, Sigma) containing or not 0.1% Triton X-100, followed by overnight incubation at 4°C with rabbit anti-human PCSK9 (1:250), mouse anti-V5 (1/500; Cat. #R960-025, Life Technologies) and mouse anti-Golgin-97 (1/500; Cat. #sc-59820, Santa Cruz Biotechnology). Specificity of endogenous PCSK9 labeling was performed by immunoadsorption with 1 μg/ml of recombinant human PCSK9 mixed with the rabbit anti-human PCSK9 antibody (1:250) in 0.1% Triton X-100/PBS and rotated overnight at 4°C prior to incubation on cells. Afterward, cells were incubated for 60 min with corresponding Alexa Fluor-conjugated secondary antibodies (Molecular Probes) and mounted in 90% glycerol containing 5% 1,4-diazabicyclo[2.2.2]octane (DABCO; Cat. #D27802, Sigma). For PCSK9-mCherry and LDLR-EGFP subcellular visualization, cells were transfected with corresponding plasmids or LDLR-EGFP expressing cells wereincubated with conditioned media containing PCSK9-mCherry. Twenty to forty-hours post-treatments, cells were washed three times with PBS and fixed with 4% paraformaldehyde/PBS for 15 min. For live-cell imaging, cells were transfected without or with PCSK9-mCherry and LDLR-EGFP or incubated with conditioned media containing PCSK9-mCherry in a CO_2_-, humidity- and temperature-controlled chamber, from which pictures were taken as indicated. Immunofluorescence or live-cell imaging analyses were performed with an Olympus FluoView FV10i or Zeiss LSM-710 confocal microscopes, respectively. The colocalization coefficient between PCSK9 or mutants thereof and Golgin-97 was measured by estimating the Pearson's correlation coefficient (r) value using ImageJ colocalization plugin (NIH).

### FRAP and iFRAP

Fluorescence recovery after photobleaching (FRAP) and inverse FRAP (iFRAP) experiments were performed with a Zeiss LSM 710 confocal microscope using a 63x 1.4 NA objective equipped with an environmental control system set to 37°C and 5% CO_2_ atmosphere. For visualization of mCherry, a 594-nm laser line (emission range detection of 600–700 nm) was used with the confocal pinhole set at 4.00 Airy units to minimize changes in fluorescence caused by the displacement of mCherry fusion proteins from the plane of focus. 4 X 10^5^ HepG2 cells were seeded to 35 mm plate containing a glass coverslip of 22 mm (MatTek). After 24h, cells were transfected with 2 μg of the different hPCSK9-mCherry constructs. For FRAP experiments, 24 h after transfection, the glass coverslip was mounted in the video confocal chamber at 37°C, whereas for iFRAP experiments, cells were pre-incubated for 2 h in media without phenol at 19.5°C to block the exit of proteins at the Golgi [42] in the presence of 200 μg/ml cycloheximide to block protein synthesis. For FRAP experiments, the whole Golgi area was photobleached using 100 scans with the 594 nm laser line at full power. To correct for changes in fluorescence efficiency attributable to mCherry proteins moving away from the plane of focus, five images were taken before the bleach. To detect the fast component of recovery after bleaching, images were taken each minute for 30 min at low laser power (20% power). No photobleaching was observed during recovery. For iFRAP experiments, the whole cytoplasm area of transfected cells, excluding the perinuclear region, were photobleached using 100 scans with the 594 nm laser line at full power. Afterward, images were collected each minute for 130 min at low laser power (20% power) to detect loss of fluorescence (exit of mCherry-tagged proteins) at the Golgi.

Quantitative analysis of FRAP (half time (t_1/2_) and mobile fraction (M_ƒ_)) was performed using easyFRAP program [43]. All recovering FRAP curves were background subtracted, normalized and fitted using the equation [44]:

$$F(t)norm=100\times \frac{\left(F(t)ROI-Fbkgd\right)}{\left(F(t)cell-Fbkgd\right)}\times \frac{\left(Fi\_cell-Fbkgd\right)}{\left(Fi\_ROI-Fbkgd\right)}.$$

All iFRAP experiments were background subtracted, corrected and normalized using the equation described below. Background fluorescence was measured in a random field outside of cells. For each time point the relative loss of total fluorescence intensity in the unbleached region of interest was calculated as [44]:

Irel=(It)/(I0)×(Tmean/Tt)

Where *It* is the average intensity of the unbleached region of interest at time point *t*, *I*0 is the average pre-bleach intensity of the region of interest and *Tmean* and *Tt* are the total mean cell intensity of the whole post-bleach period and average total cell intensity at each time of postbleach, respectively. Data were plotted as fluorescence intensity of the Golgi *versus* time. Fitting of iFRAP curves was performed using Microsoft Excel and modeled assuming second degree polynomial decay equation as [44]:

$$F(\tau )=a\tau^{2}+b\tau +c$$

Where *τ* is the time constant and *F*(*τ*) is the corresponding fluorescence intensity.

The half time (t_1/2_) was calculated from fitting equation of iFRAP curve by replacing *F*(*τ*) by *Fhalf*.

*Fhalf* can be calculated as [44]:

$$Fhalf=\frac{Fpre+Fend}{2}$$

Where *Fpre* was the initial fluorescence intensity and *Fend* the final fluorescence intensity.

Mobile fraction (M_ƒ_) was calculated as [44]:

Mf=(Fpre−Fend)/Fpre

For quantification of Golgi fluorescence intensities, we manually selected Golgi-defined regions and multiplied with a binary mask of the respective regions of interest (ROIs). For quantification of mCherry signal in HepG2 cells, the absolute Golgi fluorescence intensities per ROI were calculated by multiplying the background corrected mean Golgi pixel intensities with the number of pixels within the ROI and normalized for the number of transfected cells.

### Immunogold electron microscopy

Human liver tissue samples fixed in 4% paraformaldehyde and embedded at low-temperature in Lowicryl K4M [45] were a kind gift of Dr. Moïse Bendayan (Université de Montréal). For immunogold labeling, ultrathin (100-nm) sections mounted on nickel grids were incubated with BSA 1% for 10 min followed by overnight incubation at 4°C with rabbit anti-human PCSK9 (1:50 in BSA 1%). Tissue sections were next washed in PBS, blocked with BSA 1% for 10 min and revealed with the affinity purified secondary goat anti-rabbit IgG-gold 12nm antibody (Cat. #111-205-144, Jackson Immunoresearch) for 30 min. Sections were washed in PBS followed by distilled water and then dried, counterstained with uranyl acetate and examined using a Philips CM120 electron microscope equipped with a digital camera. Control experiments included omission of the primary antibody and resulted in the absence of specific labeling.

### Statistical analysis

Statistical comparisons were done by unpaired, two tailed t-test. P<0.05 was considered statistically significant. Error bars represent standard error to the mean (S.E.M.). All experiments were performed at least three times and representative results are shown in figures.

## Results

### Characterization of PCSK9-mCherry and LDLR-EGFP constructs

**Fig 1:**
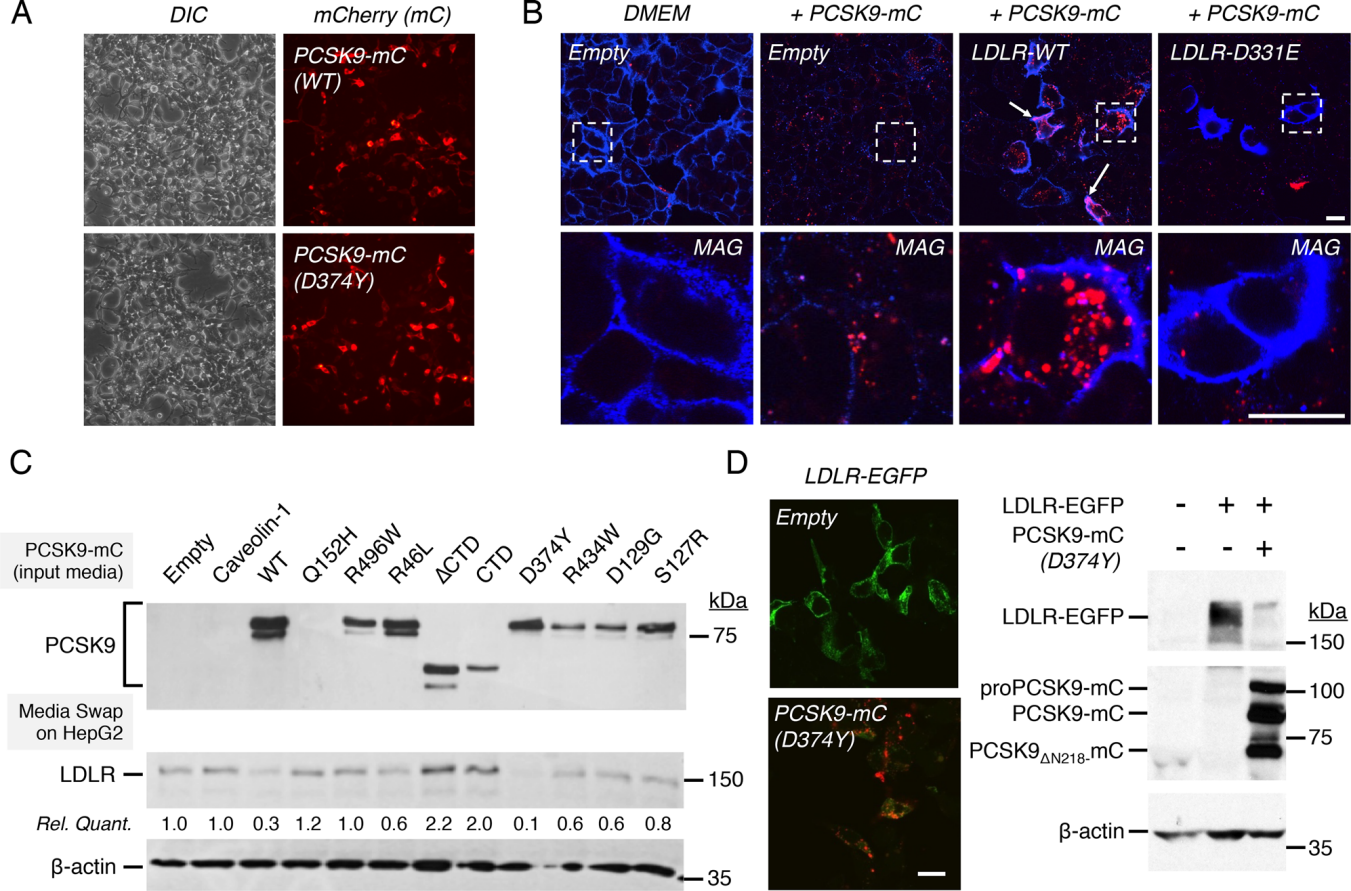
Fusion of monomeric fluorescent proteins to PCSK9 and LDLR. (A) HEK293 cells were transiently transfected with plasmids encoding WT PCSK9-mCherry (mC) or its GOF D374Y mutant and expression was analyzed by fluorescence microscopy with a 20X objective. (B) HEK293 cells were transfected without (Empty) or with WT LDLR or its EGF-A D331E mutant, incubated for 24 h (left panels) or 4 h (right panels) in DMEM or with conditioned media from PCSK9-mC transfected HEK293 cells. LDLR was revealed under non-permeabilizing conditions and protein localization was analyzed by confocal immunofluorescence microscopy. Magnified images of area within dashed lines are shown (bottom panels). *Scale bars*; 20 μm. (C) Immunoblots (IB) of conditioned media of PCSK9 natural mutants and truncation variants obtained from transiently transfected HEK293 cells (*input media*). Corresponding media were incubated overnight on HepG2 cells and LDLR and β-actin (loading control) protein levels were analyzed by IB. (D) HEK293 cells were transfected with LDLR-EGFP without (Empty) or with PCSK9 D374Y-mC and both proteins were analyzed by confocal microscopy (*left panels*) and IB (*right panels*). Scale bar = 20 μM. Data are representative of at least three independent experiments.

Fusion of fluorescent proteins to transcription factors or transmembrane and secreted proteins is a widely used tool to visualize their trafficking in cell lines and living animals [46]. Accordingly, we fused the photostable red fluorescent protein mCherry (mC; [47]) to the C-terminal of either wild-type (WT) PCSK9 or its GOF D374Y mutant. As visualized by fluorescence microscopy, PCSK9-mCherry (PCSK9-mC) chimeras were well expressed following transient transfection in HEK293 cells (Fig 1A). To ensure that fusion of mCherry does not affect the capacity of PCSK9 to induce LDLR degradation, conditioned media obtained from HEK293 cells transiently transfected with an empty vector or plasmid encoding PCSK9-mC were incubated overnight on naïve cells. Confocal microscopy data clearly showed that endogenous LDLR levels were markedly reduced following incubation with PCSK9-mC (Fig 1B; *left panels*). As expected, PCSK9-mC is internalized and located in punctate structures reminiscent of late endosomes/lysosomes [21, 22]. The internalization of PCSK9-mC is strongly enhanced following 4h incubation with cells overexpressing wild-type LDLR (Fig 1B; *middle right panel*). Interestingly, PCSK9-mC was not internalized in cells overexpressing the LDLR-D331E mutant known to prevent PCSK9 binding from biochemical studies [24], supporting the PCSK9-EGF-A interaction as the main determinant for LDLR-mediated PCSK9 endocytosis (Fig 1B; *right panel*).

We next compared the effect of natural mutations on PCSK9-mC maturation, secretion and LDLR-degrading activity (Fig 1C). HEK293 cells were transfected with WT PCSK9-mC, GOF mutants (S127R, D129G, D374Y and R496W), LOF mutants (R46L, Q152H or R434W) or caveolin-1-mC (herein used as a control) and conditioned media were incubated onto naïve HepG2 cells to evaluate endogenous LDLR degradation. With the exception of the Q152H LOF that fully blocks PCSK9 autocatalytic processing and secretion [48], WT PCSK9-mC and its mutants were relatively well secreted however at reduced levels for R434W-mC and D129G-mC mutants (Fig 1C). As compared to WT PCSK9-mC, D374Y-mC GOF mutant maintained its much greater capacity to enhanced LDLR degradation (Fig 1C). Intriguingly, it was reported that fusion of DsRed to PCSK9-ΔCTD was able to rescue its inability to induce LDLR degradation [49]. While mCherry has 87% identity to DsRed, PCSK9-ΔCTD-mC or CTD-mC remained unable to reduce LDLR protein levels in HepG2 cells (Fig 1C).

Thereafter, we fused the enhanced green fluorescent protein (EGFP) at the C-terminus of LDLR following its cytoplasmic tail. As shown by confocal microscopy and Western blot analysis, PCSK9-D374Y-mC markedly reduces LDLR levels in HEK293 cells stably expressing LDLR-EGFP both when added exogenously (Fig 1D, *left panels*) and following its transfection (Fig 1D, *right panels*). Thus, we conclude that C-terminal fusion of mCherry to PCSK9 or EGFP to LDLR did not alter their synthesis, maturation and trafficking and that PCSK9-mC is fully functional to induce LDLR-EGFP degradation both upon its overexpression in cells or *via* the extracellular pathway.

### Validation of dual fluorescence PCSK9-LDLR cell-based assay

**Fig 2:**
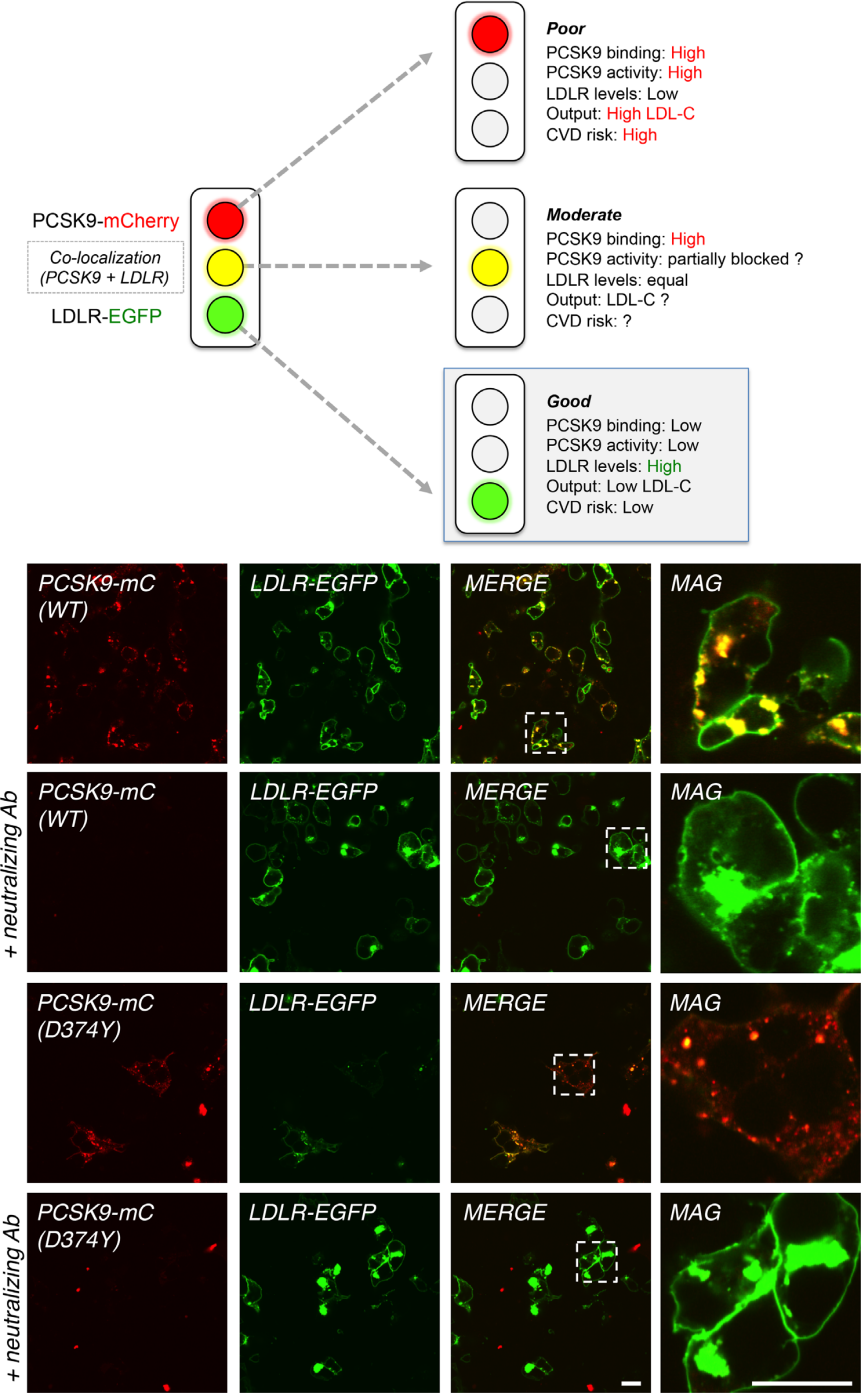
Dual fluorescence cell-based assay using PCSK9-mC and LDLR-EGFP. Schematic representation of different readouts that could be obtained from PCSK9-mC and LDLR-EGFP co-expressing cells is shown (*upper panels*). HEK293 cells were transfected with LDLR-EGFP and incubated for 4 h with WT PCSK9-mC or PCSK9 D374Y-mC containing media obtained from transfected cells without or with 4 nM anti-PCSK9 neutralizing antibody pre-incubated overnight (lower panels). Selected regions (dashed squares) were digitally zoomed 5X (magnified images, MAG). Scale bars = 20 μM. Data are representative of at least three independent experiments.

We then wanted to test our PCSK9-mC and LDLR-EGFP system, which could be used to identify small molecule inhibitors or to map important PCSK9 protein modulators by CRISPR-Cas9 sgRNA-mediated knockout libraries in large functional screens. As reference readout, control (PCSK9-mC alone), ineffective inhibitors or removal of irrelevant proteins in PCSK9-induced LDLR degradation pathway will result in low LDLR-EGFP levels and high PCSK9-mC (Fig 2; *top panel*). In the case of blockade of PCSK9 degrading activity but not its binding to LDLR, one would observe yellow labeling due to cell surface and intracellular localization of PCSK9-mC-LDLR-EGFP complex without degradation (Fig 2; *top panel*). As a third scenario, a potent PCSK9 inhibitor or removal of critical protein will give high LDLR-EGFP (green) and low PCSK9-mC signals that might be considered as a potential lipid-lowering therapy or class of molecules (Fig 2; *top panel*). To validate our dual fluorescence PCSK9-LDLR cell-based assay, conditioned media from HEK293 cells overexpressing WT PCSK9-mC or its GOF D374Y mutant were incubated for 4 h with LDLR-EGFP expressing HEK293 cells in the absence or presence of a PCSK9 neutralizing antibody. Confocal microscopy data showed that the PCSK9 neutralizing antibody strongly prevents binding of WT and D374Y PCSK9-mC (Fig 2; *lower left panels*) to LDLR-EGFP and protects LDLR from PCSK9-induced degradation (Fig 2; *lower middle left panels*, green), establishing a simple, specific and cost-effective cell-based assay.

### Importance of PCSK9 CTD for LDLR-mediated PCSK9 endocytosis

**Fig 3:**
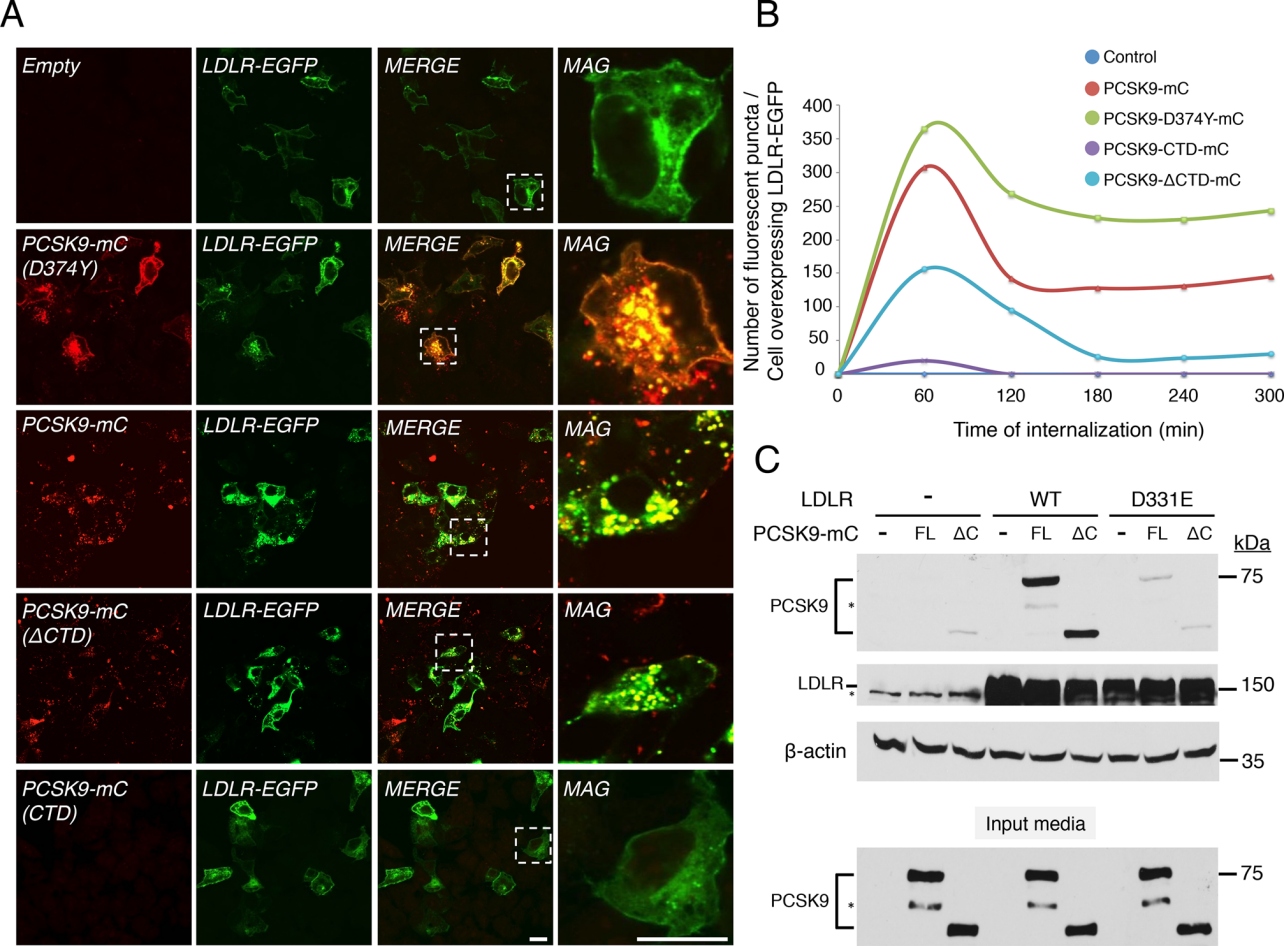
PCSK9 CTD is required for efficient LDLR-mediated endocytosis of PCSK9. (A) HepG2 cells transfected with LDLR-EGFP were incubated 5h with conditioned media obtained from HEK293 cells transected without (Empty) or with PCSK9 WT-, D374Y-, ΔCTD- or CTD-mC. PCSK9-mC constructs and LDLR-EGFP were analyzed by live-cell confocal microscopy. Selected regions (dashed squares) were digitally zoomed 5X (MAG). Scale bars = 20 μM. (B) Quantification of the number of intracellular red fluorescent puncta per cell expressing LDLR-EGFP for each PCSK9 mCherry fusion construct described in (A) after incubation for 0, 60, 120, 180, 240 and 300 min. PCSK9 WT-mC (n = 37 cells), D374Y-mC (n = 62 cells), ΔCTD-mC (n = 31 cells) or CTD-mC (n = 52 cells). (C) HEK293 cells were transfected without (-) or with WT LDLR or its EGF-A D331E mutant and incubated for 6 h with DMEM alone (-) or with normalized conditioned media obtained from FL or ΔCTD PCSK9-mC transfected cells (input media). Cell-associated PCSK9-mC, LDLR and β-actin (loading control) protein levels were analyzed by IB. Data are representative of at least three independent experiments.

We then took advantage of this cell-based assay to deepen our knowledge on the role(s) of PCSK9 CTD, the effects of natural mutants and PCSK9-LDLR trafficking dynamics. While PCSK9 CTD is required to induce LDLR degradation (PCSK9-ΔCTD; Fig 1C, [33]), it was shown that its deletion does not perturb PCSK9-LDLR complex formation [22, 33, 50]. We first tested the importance of the CTD for LDLR-mediated PCSK9 endocytosis by incubating conditioned media containing GOF PCSK9 D374Y-mC, WT PCSK9-mC, PCSK9-ΔCTD-mC or CTD-mC alone for 5 h with human hepatoma HepG2 cells overexpressing LDLR-EGFP. As depicted by confocal microscopy, GOF PCSK9 D374Y-mC strongly co-localized with LDLR-EGFP at the cell surface and in intracellular punctate structures reminiscent of late endosomes/lysosomes (Fig 3A; [22]). As compared to full-length (FL) PCSK9-mC, PCSK9-ΔCTD-mC and CTD-mC alone were less or not endocytosed, respectively (Fig 3A). As shown in Fig 3B, quantification of WT PCSK9 and D374Y GOF mutant internalization in LDLR-EGFP expressing cells over time showed that both were efficiently endocytosed, ~2-fold the amount of PCSK9-ΔCTD at 60 min. For all constructs, the number of fluorescent puncta attained a peak at ~60 min and a plateau was reached at ~180 min where WT PCSK9-mC and PCSK9 D374Y-mC fluorescent puncta were ~5- and 9-fold higher, respectively, than PCSK9-ΔCTD-mC. One possible explanation could be due to reduce binding affinity of PCSK9-ΔCTD-mC to LDLR as compared to FL PCSK9-mC. Accordingly, HEK293 cells were transiently transfected for 48 h with either WT or the PCSK9-binding defective LDLR-D331E mutant and incubated 6 h without (-) or with normalized FL or PCSK9-ΔCTD-mC conditioned media (Fig 3C). Western blot analyses revealed similar cellular association of FL and PCSK9-ΔCTD-mC in WT, and not in D331E, LDLR-overexpressing cells. Therefore, we concluded that PCSK9 CTD increases LDLR-mediated PCSK9 endocytosis without significantly affecting its binding to LDLR-EGF-A domain.

### Live-cell imaging analysis of PCSK9-mC and LDLR-EGFP trafficking

**Fig 4:**
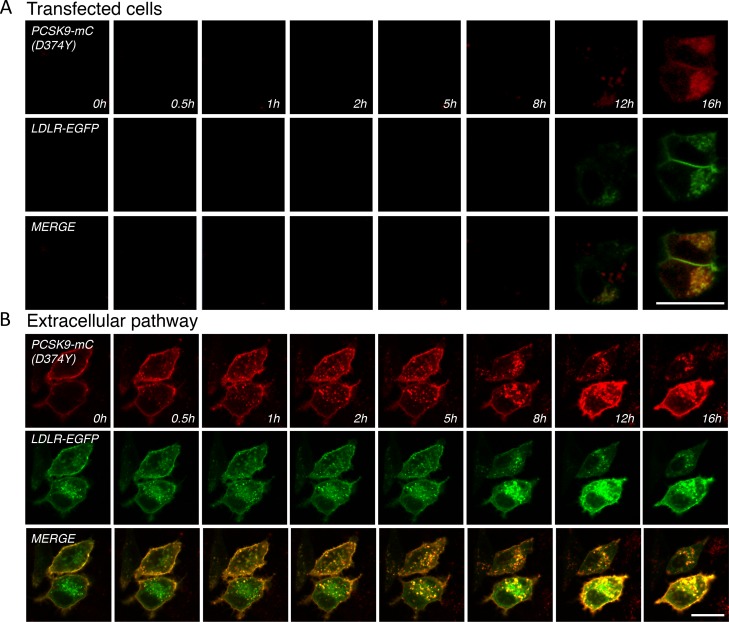
Live-cell imaging of PCSK9-D374Y-mC and LDLR-EGFP trafficking. HepG2 cells were transfected with LDLR-EGFP together with D374Y PCSK9-mC (A; transfected cells, see also S1 Video) or incubated with PCSK9 D374Y-mC conditioned media (B; extracellular pathway, see also S2 Video). Images were extracted from confocal microscopy time-lapse movies. Data are representative of at least three independent experiments. *Scale bar*, 20 μm.

To gain insights into PCSK9 and LDLR intracellular dynamics, PCSK9 D374Y-mC and LDLR-EGFP cDNAs were co-transfected in HepG2 cells mounted in a CO_2_-, humidity- and temperature-controlled chamber. Live-cell images were taken every 5 min for 16 h using a Zeiss LSM710 confocal microscope (Fig 4A). Following transfection, it took ~12 h to detect the appearance of PCSK9-D374Y-mC and LDLR-EGFP that were co-localized in intracellular compartments but with almost no detectable signal at the cell surface (Fig 4A and S1 Video). The weak signal that was observed might be attributable to the strong capacity of intracellular PCSK9-D374Y-mC to induce LDLR-EGFP degradation (see Fig 1D; *right panels*). To examine the kinetic of PCSK9 endocytosis, PCSK9 D374Y-mC was incubated with LDLR-EGFP expressing HepG2 cells and imaged every 5 min for 16 h as described above. In accordance with previous live-cell analysis [51], PCSK9 D374Y-mC rapidly bound (< 10 min) to cell surface LDLR-EGFP and internalized (< 30 min) in complex into late endocytic punctate structures (Fig 4B and S2 Video, [22]). Confocal microscopy revealed two PCSK9 D374Y-mC responsive HepG2 cell populations depending on levels of LDLR-EGFP expression (Fig 4B and S2 Video). In high cell surface LDLR-EGFP expressing cells (Fig 4B, upper cell), exogenous PCSK9 D374Y-mC efficiently and gradually induced LDLR degradation with almost complete internalization of LDLR 8 h following incubation. In low cell surface and high intracellular LDLR-EGFP expressing cells (*bottom cell*), we also observed a similar elevated rate of PCSK9 D374Y-mC internalization, which colocalized and accumulated with LDLR-EGFP in perinuclear lysosome-like organelles. Interestingly, we also observed a perinuclear inaccessible pool of LDLR-EGFP that did not show colocalization with internalized PCSK9 D374Y-mC (Fig 4B and S2 Video).

**Fig 5:**
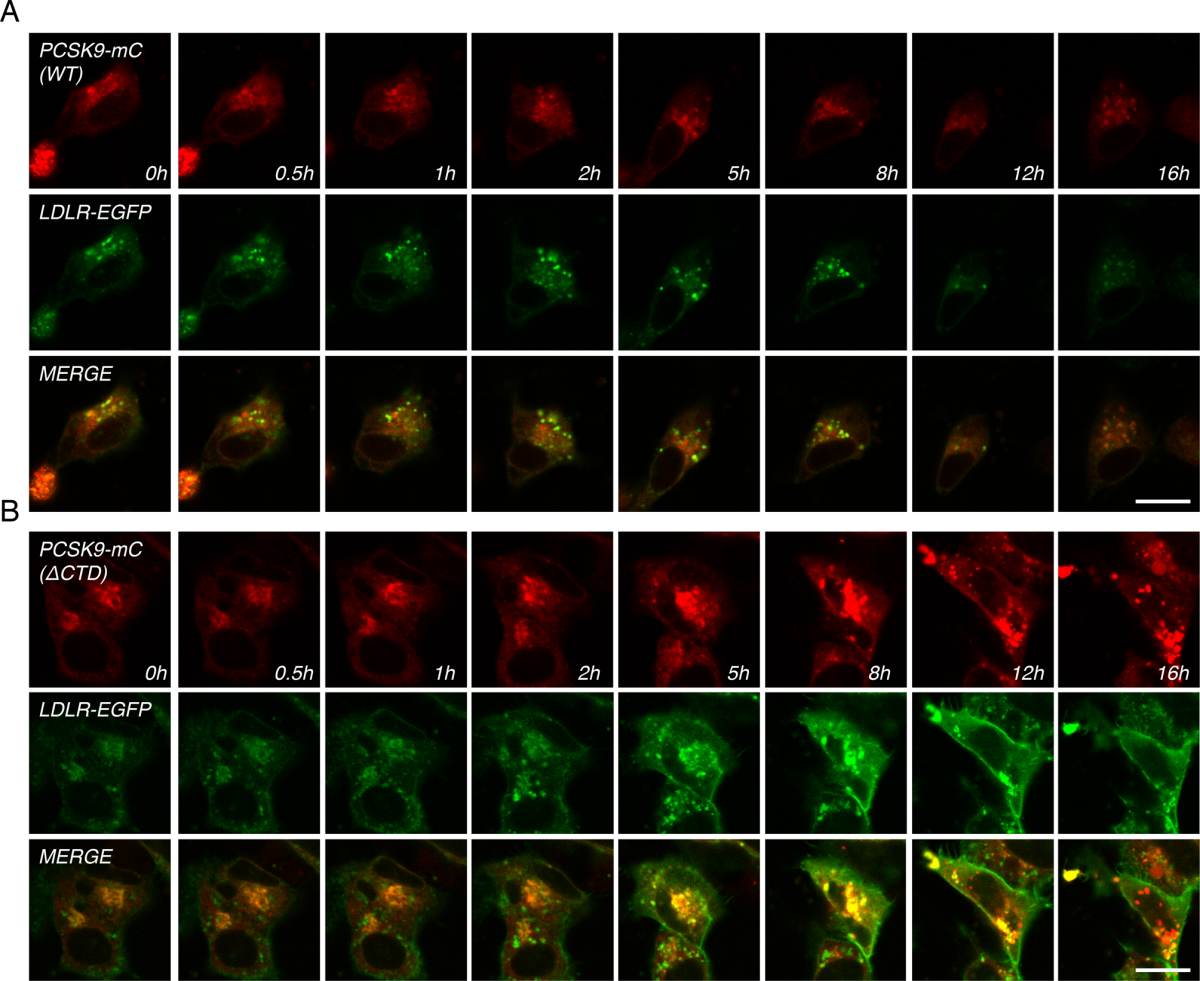
Live-cell imaging of WT PCSK9, PCSK9-ΔCTD-mC and LDLR-EGFP trafficking. HepG2 cells were co-transfected with LDLR-EGFP together with (A) WT PCSK9-mC (see S3 Video) or (B) WT PCSK9-ΔCTD (see S4 Video). Images were extracted from confocal microscopy time-lapse movies. Data are representative of at least three independent experiments. *Scale bar*, 20 μm.

PCSK9 CTD domain potentiates LDLR-mediated internalization of PCSK9 (Fig 3) and degradation of LDLR (Fig 1C). Therefore, we studied the intracellular trafficking of PCSK9-ΔCTD-mC and LDLR-EGFP in HepG2 cells 18 h following transfection by live-cell confocal microscopy (Fig 5 and S3 and S4 Videos). In contrast to WT PCSK9-mC (Fig 5A and S3 Video), PCSK9-ΔCTD-mC strongly accumulated and co-localized with LDLR-EGFP in punctate structures (Fig 5B and S4 Video) and failed to induce LDLR-EGFP degradation (Figs 1C and 5B and S4 Video). This suggests that PCSK9 CTD is not important for intracellular LDLR binding but is rather needed to induce LDLR degradation likely *via* its proposed direct interaction with LDLR [33–35] and/or by allowing a final trafficking step towards lysosomes.

### Subcellular localization of PCSK9 in the TGN depends on its CTD

**Fig 6:**
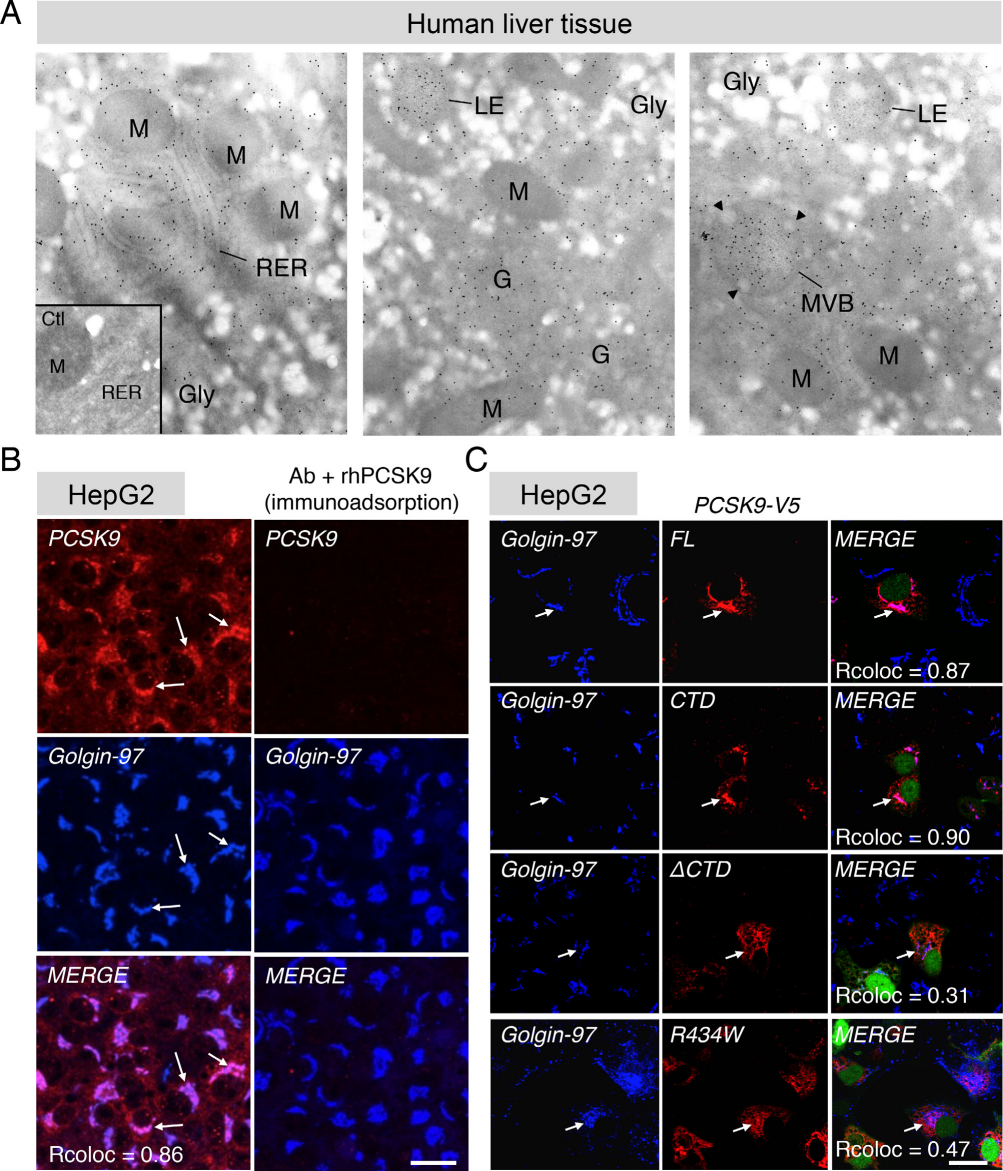
PCSK9 localizes at the TGN *via* its CTD. (A) Ultrastructural localization of PCSK9 in normal human liver tissue sections shows the immunogold labeling over the rough endoplasmic reticulum (RER; left panel), Golgi apparatus (G), mulitvesicular bodies (MVB) and late endosomes (LE) (middle and left panels) of hepatocytes. Arrowheads denotes inward budding vesicles that are characteristic of MVBs (right panel). Note the presence of very few gold particles over mitochondria (M) and glycogen (Gly) indicating the specificity of the labeling. Omission of the primary antibody resulted in the absence of specific labeling (control experiment, Ctl; inset). Magnification X 25000. (B) Co-localization of endogenous PCSK9 with the TGN marker Golgin-97 as visualized by confocal microscopy (*left panels*, *white arrows*). Specificity of PCSK9 immunolabeling was tested following overnight pre-incubation of the anti-PCSK9 Ab with 1 μg/ml recombinant human PCSK9 (*right panels*, *immunoadsorption*). (C) HepG2 cells were transfected with V5-tagged FL PCSK9, CTD, ΔCTD or LOF R434W mutant and co-localization with Golgin-97 was analyzed by confocal microscopy. (B, C) Colocalization was quantified using Pearson’s correlation coefficient (Rcoloc) (minimum of 10 cells were analyzed). Data are representative of at least three independent experiments. *Scale bars*, 20 μm.

**Fig 7:**
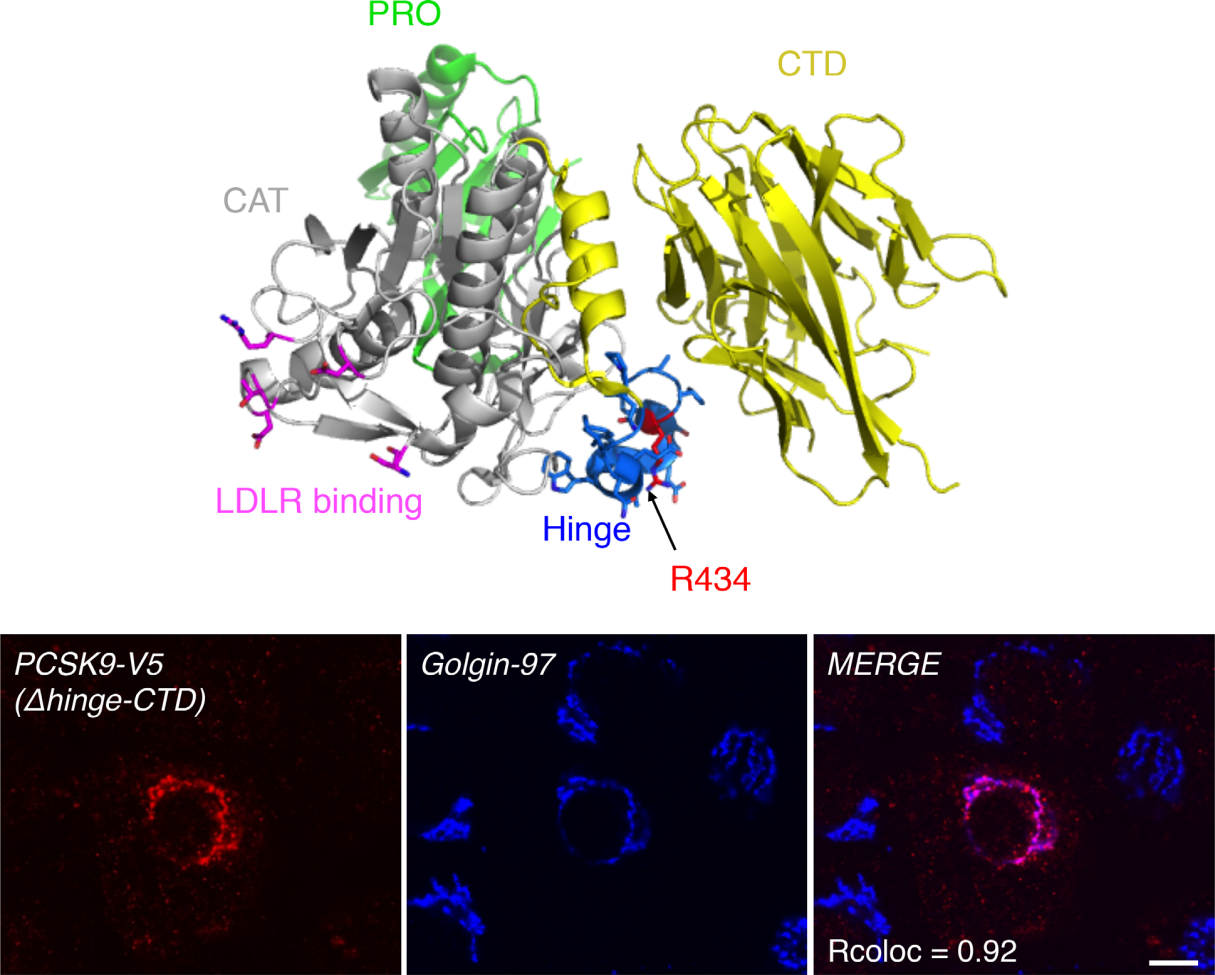
Deletion of PCSK9 hinge region does not affect CTD localization at the TGN. PCSK9 molecular structure was extracted from PDB 2P4E using MacPymol software. Prodomain (PRO), catalytic domain (CAT) and its LDLR-interacting residues (in pink), hinge region in blue and position R434 in red (site of LOF R434W) and C-terminal domain (CTD) are indicated. HepG2 cells were transfected with V5-tagged ΔHinge-CTD (Δaa 422-439-CTD), immunolabeled with the anti-V5 and anti-Golgin-97 antibodies and their localization was analyzed by confocal microscopy (*lower panels*). Colocalization was quantified using Pearson’s correlation coefficient (Rcoloc) (minimum of 10 cells were analyzed). Data are representative of at least three independent experiments. *Scale bar*, 10 μm.

To better understand to role of PCSK9 CTD, we first studied the intracellular localization of PCSK9 in human liver sections (Fig 6A). Post-embedding immunogold electron microscopy revealed that PCSK9 is present specifically in the rough ER (RER) (Fig 6A, left panel), *Golgi* apparatus (G) (Fig 6A, center panel), mulitvesicular bodies (MVB) and late endosomes (LE) of human hepatocytes (Fig 6A, right panel). In human HepG2 cells, in addition to its colocalization with RER markers [15] (not shown), endogenous PCSK9 was strongly colocalized with the TGN marker Golgin-97 and accumulated in this compartment (Fig 6B). Furthermore, FL PCSK9 and the CTD alone, but not PCSK9-ΔCTD or PCSK9 R434W natural LOF mutant that exhibit mainly an ER-like localization, were colocalized with Golgin-97, emphasizing the critical role of PCSK9 CTD for its localization at the TGN (Fig 6C). The PCSK9 CTD domain is linked to the catalytic domain through an exposed hinge region where PCSK9 LOF mutant R434W, which fails to concentrate at the TGN, is positioned (Fig 7; *top panel*, residues 422–439). To test the importance of the hinge region in PCSK9 trafficking, we analyzed the subcellular localization of the CTD without the hinge region at its N-terminus following signal peptide (ΔHinge-CTD). In contrast with PCSK9-ΔCTD and similar to the CTD (Fig 6C), the ΔHinge-CTD alone colocalized with the TGN marker Golgin-97 in HepG2 cells (Fig 7; *lower panels*), suggesting that the hinge region does not participate to the CTD-mediated PCSK9 localization at the TGN.

**Fig 8:**
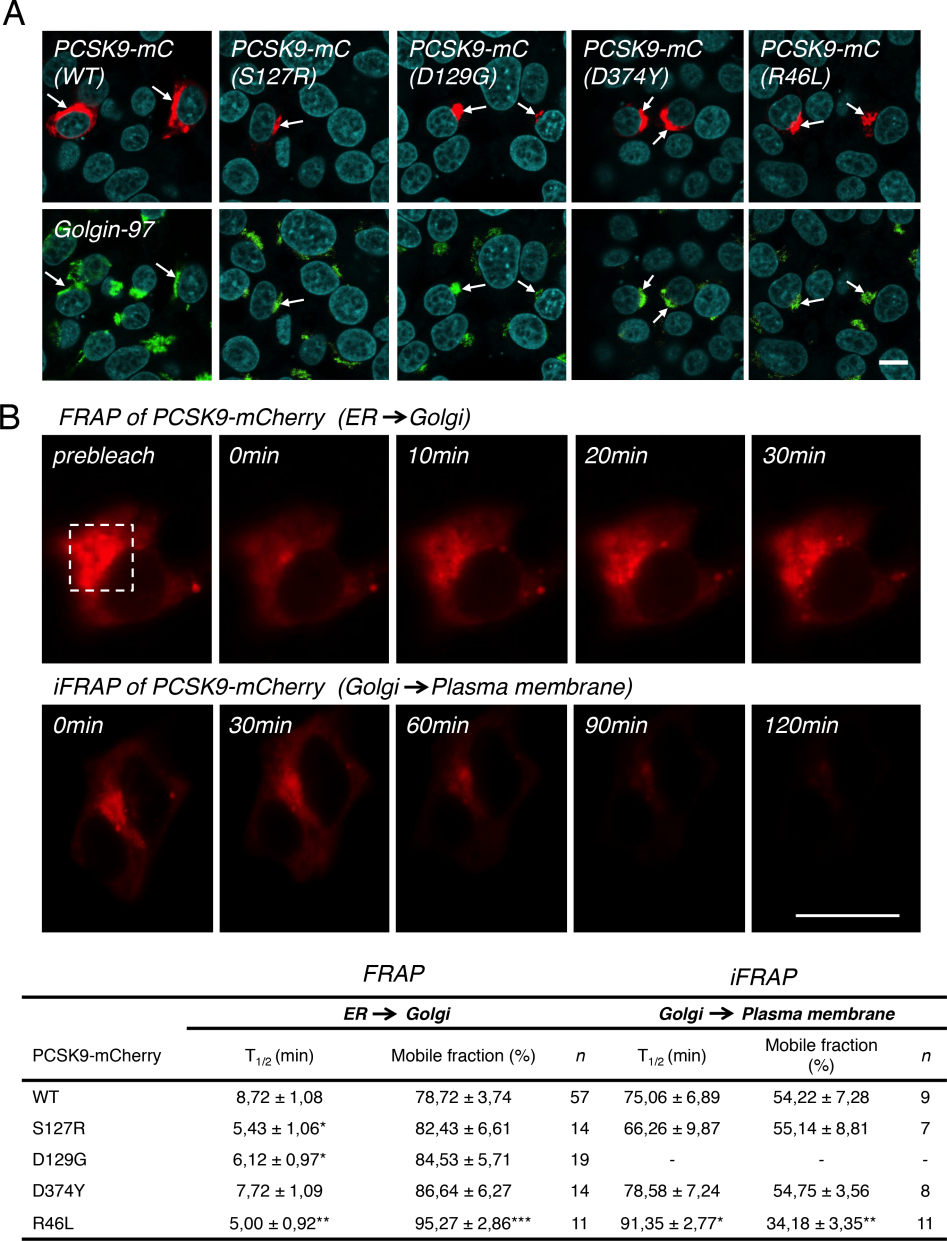
Effect of PCSK9 natural mutants on TGN localization and trafficking dynamics by FRAP and iFRAP. (A) HepG2 cells were transfected with PCSK9-mC WT, GOF S127R, D129G, D374Y or LOF R46L mutants (red) and TGN localization was determined by colocalization (arrows) with Golgin-97 (green) and analysis by confocal microscopy. Nuclei were labeled with TO-PRO-3 Iodide (cyan). (B) Half time (T_1/2_; min) and mobile fraction (%) calculated from FRAP (ER ➜ Golgi) and iFRAP (Golgi ➜ plasma membrane) experiments in living HepG2 cells for corresponding PCSK9-mC constructs shown in (A). Dashed square represent typical TGN localization of PCSK9-mC before bleach for which fluorescence recovery after photobleaching (FRAP) values were obtained (FRAP). Inverse FRAP (iFRAP) studies in the presence of 200 μg/ml cycloheximide to block protein synthesis (as described in Materials and Methods) were performed for corresponding PCSK9-mC shown in (A). For each PCSK9-mC constructs, mobile fraction (%) at the TGN from FRAP and iFRAP were calculated as described in *Materials and* Methods. Data are representative of at least three independent experiments are shown as the mean ± S.E.M. Statistical significance: *p ≤ 0.05, **p ≤ 0.01, ***p ≤ 0.001. *Scale bars*, 10 μm.

We next decided to further investigate the effects of GOF and LOF natural mutations on PCSK9 intracellular trafficking with a focus on TGN localization. At steady state, WT PCSK9-mC, GOF S127R, D129G, D374Y and LOF R46L mutants, but not R434W (Fig 6C), all colocalized with Golgin-97 at the TGN (Fig 8A). To determine if these constructs accumulate to the same extent at the TGN, we measured their relative fluorescence intensities in the perinuclear region in live cells. As compared to WT PCSK9-mC, quantification of equivalent region of interest revealed a higher intensity of the GOF D129G at the TGN (+49%), while GOF S127R (-34%) and LOF R46L (-28%) mutants showed lower fluorescence intensities (S1 Fig). The D374Y GOF mutant and CTD-mC showed no significant difference in TGN fluorescence intensities as compared to WT PCSK9 (S1 Fig).

### Trafficking dynamics of PCSK9 and mutants thereof

To determine if GOF or LOF mutations modify the intracellular trafficking of PCSK9 in the secretory pathway, we analyzed the protein mobility of WT PCSK9-mC, GOF mutants S127R, D129G and D374Y as well as R46L LOF mutant by fluorescence recovery after photobleaching (FRAP; Fig 8B, *top panels*). Live-cell confocal analysis following photobleaching of the Golgi pool of fluorescence of WT PCSK9-mC revealed a half time fluorescence recovery from the nonbleached ER pool (i.e. transport from the ER towards the Golgi apparatus) of 8.72 ± 1.08 min (Fig 8B). As compared to WT PCSK9-mC, GOF mutations S127R (5.43 ± 1.06 min), a mutant with impaired proPCSK9 to PCSK9 conversion [52, 53], and D129G (6.12 ± 0.97 min) or LOF mutation R46L (5.00 ± 0.92 min) were significantly faster to reach the Golgi (Fig 8B). However, the R46L mutant had a significantly higher mobile fraction as compared to WT PCSK9-mC (95.27% ± 2.86 *vs* 78.72% ± 3.74) indicating increased retention of the mutant at the Golgi (Fig 8B).

Thereafter, we analyzed the trafficking dynamics of GOF or LOF mutations by inverse FRAP (iFRAP) that allowed measuring exit and clearance time of a given PCSK9-mC construct from the TGN toward the cell surface (Fig 8B; *lower panels*). Twenty-four hours following transfection, HepG2 cells were incubated 2 h with 200 μg/ml cycloheximide to block *de novo* protein synthesis and at 19.5°C to allow retention of PCSK9-mC at the TGN. The whole cytoplasm area of transfected cells, excluding the perinuclear region, was photobleached and images were collected to detect loss of fluorescence at the Golgi. WT PCSK9-mC had a half time of 75.06 ± 6.89 min to exit the TGN. Its mobile fraction was 54.22% ± 7.28 indicating that a significant proportion of PCSK9 resides at the TGN (Fig 8B; iFRAP), which is in accordance with our observations that PCSK9 concentrates at the TGN at steady state (Fig 6). No significant difference was observed both for the exit or mobile fraction at the TGN for S127R, D129G and D374Y GOF mutants as compared to WT PCSK9-mC (Fig 8B). However, the R46L LOF mutant was found to be much slower to exit the TGN (T_1/2_, 91.35 ± 2.77 min) and had a much lower mobile fraction (34.18% ± 3.35) again indicating increased retention in the TGN (Fig 8B). Altogether, these results are indicative of an impaired post-Golgi trafficking for the R46L mutant compared with WT PCSK9-mC, which could explain the decreased activity of the R46L mutant in inducing LDLR degradation [54].

### Lysosomal targeting of PCSK9-ΔCTD rescues its capacity to induce LDLR degradation

**Fig 9:**
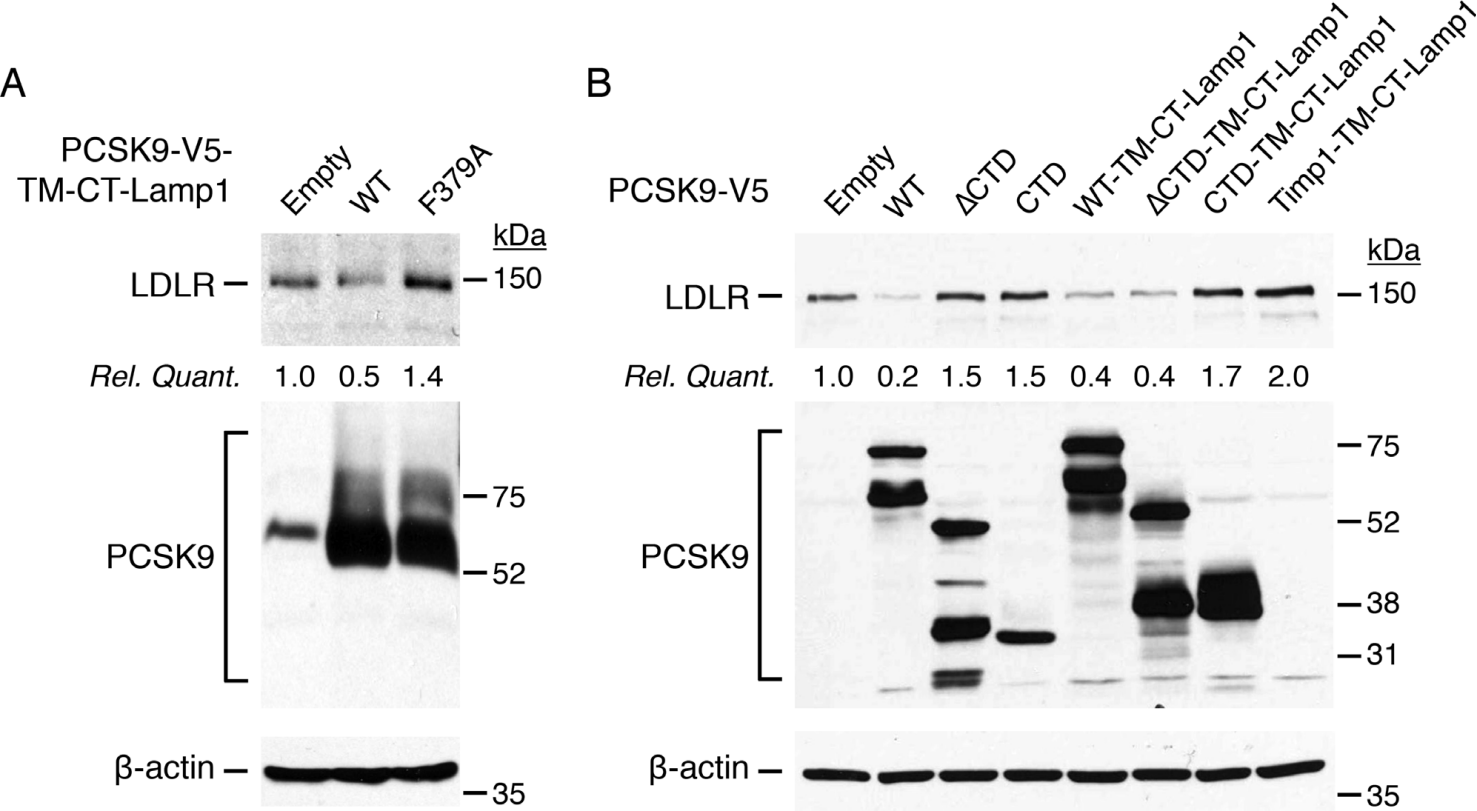
Lysosomal targeting of PCSK9-ΔCTD bypasses the need of CTD to induce LDLR degradation. (A-B) HepG2 cells were transfected without (Empty) or with V5-tagged PCSK9 full-length (FL), ΔCTD, CTD alone or PCSK9-TM-CT-Lamp1 chimeras (FL, F379A, ΔCTD, CTD) or Timp1-TM-CT-Lamp1 (herein used as control). Forty-eight hours post-transfection, LDLR, PCSK9 and β-actin (loading control) protein levels were analyzed by IB. Data are representative of at least three independent experiments

Although there is a positive correlation between PCSK9 CTD and LDLR-mediated endocytosis of PCSK9 (Fig 3), PCSK9 localization at the TGN (Figs 6 and 7) and PCSK9 capacity to induce LDLR degradation (Figs 1C and 5; [33]), the exact role(s) of the CTD remain elusive. It has been proposed that PCSK9 CTD directly interacts with the LDLR ligand domain at endosomal acid pH [34] allowing ectodomain cleavage of the LDLR and its degradation [35]. In addition, the freely accessible PCSK9 CTD may be needed for proper intracellular trafficking of the PCSK9-LDLR complex, possibly *via* binding to amyloid precursor-like protein 2 (APLP2; [18]) or yet unidentified protein(s) [16, 36], towards late endocytic compartments. We previously showed that fusion of the transmembrane (TM) and cytosolic tail (CT) of the lysosomal-associated membrane protein 1 (Lamp1) to PCSK9 greatly increases its targeting to late endosomal/lysosomal compartment and its capacity to induce degradation of LDLR and its family members in various cell lines [55]. Thus, we transfected HepG2 cells with TM-CT-Lamp1 chimeras fused to WT PCSK9 or its LDLR-binding defective F379A mutant [11], PCSK9-ΔCTD, CTD alone or tissue inhibitor of metalloproteinase 1 (Timp1, herein used as negative control). As predicted, lysosomal targeting of WT PCSK9, but not PCSK9-F379A, induced LDLR degradation *via* direct PCSK9-LDLR complex formation (Fig 9A). While overexpression of soluble ΔCTD and CTD did not decreased LDLR levels as compared to WT PCSK9, fusion of TM-CT-Lamp1 to PCSK9-ΔCTD (PCSK9-ΔCTD-TM-CT-Lamp1), but not to CTD or Timp1, completely rescued the capacity of PCSK9-ΔCTD to promote LDLR degradation at similar potency of soluble FL PCSK9 and FL PCSK9-TM-CT-Lamp1 (Fig 9B). These results show that PCSK9 CTD is dispensable if PCSK9 is artificially targeted to late endocytic compartments, suggesting a role of the CTD for proper intracellular sorting of the PCSK9-LDLR complex.

## Discussion

While the use of PCSK9 inhibitors is well documented [56], the cellular dynamics and basic biology of PCSK9 are still not fully understood. Therefore, we fused monomeric fluorescent mCherry to PCSK9 and EGFP to LDLR C-terminal tails to perform live-cell imaging and structure/function studies. We first showed that LDLR-EGFP is well expressed and localized at the cell surface and that PCSK9-mC retains its capacity to induce both LDLR and LDLR-EGFP degradation either *via* overexpression or the extracellular pathway (Fig 1). Similar to other LDLR ligands [57], following its incubation on hepatic cells PCSK9-mC efficiently binds to LDLR-positive microdomains at the cell surface and complexes are rapidly internalized in endocytic vesicles (Fig 4; *lower panels* and S2 Video). As expected [24], disruption of Ca^2+^ binding to the LDLR-EGF-A domain completely prevents PCSK9-mC cell surface binding and internalization (Figs 1B and 3C; LDLR D331E *vs* WT), thus reinforcing the major role of the EGF-A domain for LDLR-mediated PCSK9 endocytosis.

Biochemical and co-crystallization studies have previously shown that PCSK9 CTD is not directly involved in LDLR-EGF-A complex formation but is essential to induce LDLR degradation [33, 50]. Herein, we showed that PCSK9-ΔCTD-mC is endocytosed, albeit at lower levels than FL PCSK9-mC, in LDLR-EGFP expressing cells (Fig 3), but fails to induce LDLR degradation (Fig 1C). However, in a previous study Holla et al. showed that replacement of the CTD domain with DsRed, another small fluorescent protein similar to mCherry, rescued the function PCSK9-ΔCTD and induced LDLR degradation [49]. We surmise that self-association and charges may be important features of the PCSK9 CTD since DsRed is known to form higher-order aggregates and to be positively charged [58] while mCherry has a more neutral charge and does not self-associate [47]. To date, it still remains puzzling how PCSK9 CTD governs LDLR degradation. In a screening experiment, it has been proposed that sorting of the PCSK9-LDLR complex towards late endocytic compartments for degradation is mediated by binding of the PCSK9 CTD to APLP2 at the cell surface [18]. However, the importance of APLP2 *in vivo* remains controversial since hepatic LDLR protein levels are unaffected in *Aplp2*^-/-^ mice and that exogenous addition of PCSK9 induce LDLR degradation in *Aplp2*-deficient primary hepatocytes [16].

PCSK9 is known to target LDLR for degradation both *via* extra- and intracellular pathways [19, 21]. More precisely, the M2 module of the CTD is essential for extracellular PCSK9 to induce LDLR degradation but dispensable for the intracellular pathway [59], likely suggesting a distinctive trafficking role for this subdomain. In the present study, immunogold electron microscopy showed that endogenous PCSK9 localizes in the ER, *Golgi* apparatus, multi-vesicular bodies and late endosomes in human liver tissue (Fig 6A) and in the ER and TGN in immortalized human liver HepG2 cells (Fig 6B). Moreover, FL PCSK9 or its CTD alone, but not PCSK9-ΔCTD, were present at the TGN emphasizing an essential role of the CTD for PCSK9 intracellular localization (Fig 6C). Our results showed that, similar to endogenous PCSK9, PCSK9 GOF (S127R, D129G, and D374Y) and LOF (R46L) mutants were found accumulated at the TGN (Fig 8A). LOF mutation R434W within the hinge region of PCSK9, linking the catalytic domain to the CTD (Fig 7; *top panel*), abolished the localization of PCSK9 at the TGN (Fig 6C). However, we found that the hinge region was not directly involved in intracellular retention of PCSK9 CTD (Fig 7; *lower panels*, CTD *vs* ΔHinge-CTD), indicating that the R434W mutation impedes PCSK9 retention at the TGN possibly by altering CTD conformation. Interestingly, live-cell imaging revealed that PCSK9-ΔCTD-mC co-localized with LDLR-EGFP in intracellular vesicles without inducing its degradation (Fig 5B and S4 Video). However, fusion of the TM-CT of Lamp1 to PCSK9-ΔCTD was able to rescue its ability to induce LDLR degradation likely through bypassing a crucial CTD-binding factor required for late trafficking and/or degradation of PCSK9-LDLR complex (Fig 9). These results support that PCSK9 binding to LDLR is not sufficient for its degradation and that once in the TGN or in endosomes PCSK9 possibly interacts with a co-receptor through its CTD, which would lead LDLR to the lysosomes.

PCSK9 has emerged as a critical player for the regulation of plasma LDL-cholesterol homeostasis [23]. Indeed, GOF [5] or LOF [6] mutations in PCSK9 directly correlate with levels of circulating atherogenic LDL particles and risks of CVD. Interestingly, several mutations in PCSK9 were found external to its LDLR interacting domain [60], for which there is little information on the mechanism by which they influence LDLR and circulating LDL levels. Therefore, we sought to gain insight into the mechanism of the PCSK9-induced LDLR degradation by analyzing the subcellular localization and trafficking dynamics of PCSK9 and its natural mutants. The intracellular mobility of WT PCSK9-mC and of its GOF/LOF mutations was determined by FRAP experiments in living human HepG2 cells. Compared to WT PCSK9, all mutants (S127R, D129G and R46L), excepted for PCSK9-D374Y, had increased protein mobility from the ER to Golgi (>35% compared to WT; Fig 8B). This could help explain the increased activity of GOF mutations S127R and D129G, which exit the ER and reach the TGN faster than WT PCSK9, where they may bind and direct the LDLR to the lysosomes more efficiently. Data from iFRAP experiments revealed that, similar to WT PCSK9, S127R and D374Y accumulate at the TGN and that only half of this cellular fraction leaves this compartment and is secreted (Fig 8B). These results suggest that S127R and D374Y mutations modify the trafficking of PCSK9 from the ER to the Golgi but not its exit from the TGN to the plasma membrane. It was previously shown that PCSK9 is phosphorylated by a Golgi casein kinase-like kinase in the prodomain at position S47 and in the CTD at S688 and that LOF R46L significantly reduced phosphorylation at S47 [61]. Interestingly, our data showed that LOF mutation R46L, which had the highest mobility between the ER and the TGN, was significantly slower to exit the TGN (T_½_ = 91.35 ± 2.77 *vs* 75.06 ± 6.89 min) and had a decreased mobile fraction compared to WT PCSK9 (Fig 8B). Thus, our results suggest that TGN retention of PCSK9 LOF R46L mutation, and possibly the lower phosphorylation at S47, may reduce its secretion and/or impede the targeting and degradation of LDLR into late endocytic compartments and thus consequently increase LDL clearance in the bloodstream.

In the present study, we developed a dual fluorescence PCSK9-LDLR cell-based assay and studied their intracellular dynamics under different parameters. We demonstrated an important role of the PCSK9 CTD for its LDLR-dependent internalization and TGN localization and underline an essential role as a late intracellular trafficking determinant. Finally, this cell-based assay, which was validated using a blocking anti-PCSK9 monoclonal antibody, could be used in a high-throughput screening to identify biological or small molecule inhibitors or in a genome-wide shRNA or Cas9-CRISPR functional studies.

## Supporting Information

S1 Fig Relative fluorescence intensities of PCSK9-mC constructs at the TGN. HepG2 cells were transfected with FL PCSK9-mC (n = 32), GOF S127R (n = 31), D129G (n = 22), D374Y (n = 25), LOF R46L (n = 41) or CTD alone (n = 53) and Golgi fluorescence intensities were measured as described in Material and Methods. Data are shown as the mean ± S.E.M. relative to WT PCSK9. *p ≤ 0.05, **p ≤ 0.01.
(TIF)

S1 Video Live-cell imaging of co-expressed GOF D374Y PCSK9-mC and LDLR-EGFP. HepG2 cells were transfected with LDLR-EGFP together with GOF D374Y PCSK9-mC. After mounting cells in a temperature-, humidity- and CO_2_-controlled chamber, images were acquired every 5 min for a total of 16h using a Zeiss LSM 710 confocal microscope. This video is representative of at least three independent experiments.
(MOV)

S2 Video Live-cell imaging of LDLR-EGFP-mediated GOF D374Y PCSK9-mC internalization. HepG2 cells were transfected with LDLR-EGFP, mounted in a temperature-, humidity- and CO_2_-controlled chamber, and incubated with conditioned media containing PCSK9 GOF D374Y-mC. Live-cell images were acquired every 5 min for a total of 16h using a Zeiss LSM 710 confocal microscope. This video is representative of at least three independent experiments.
(MOV)

S3 Video Live-cell imaging of co-expressed WT PCSK9-mC and LDLR-EGFP. HepG2 cells were transfected with LDLR-EGFP together with WT PCSK9-mC and mounted in a temperature-, humidity- and CO_2_-controlled chamber. Live-cell images were acquired 16h post-transfection every 5 min for a total of 16h using a Zeiss LSM 710 confocal microscope. This video is representative of at least three independent experiments.
(MOV)

S4 Video Live-cell imaging of LDLR-EGFP and PCSK9-ΔCTD-mC intracellular trafficking. HepG2 cells were transfected with LDLR-EGFP together with PCSK9-ΔCTD mC and mounted in a temperature-, humidity- and CO_2_-controlled chamber. Live-cell images were acquired 16h post-transfection every 5 min for a total of 16h using a Zeiss LSM 710 confocal microscope. This video is representative of at least three independent experiments.
(MOV)
