# Supplementary figures and images for: Trafficking Dynamics of PCSK9-Induced LDLR Degradation: Focus on Human PCSK9 Mutations and C-Terminal Domain
Source: PLoS One. 2016 Jun 9;11(6):e0157230. doi: 10.1371/journal.pone.0157230 (PMC4900664; doi:10.1371/journal.pone.0157230)

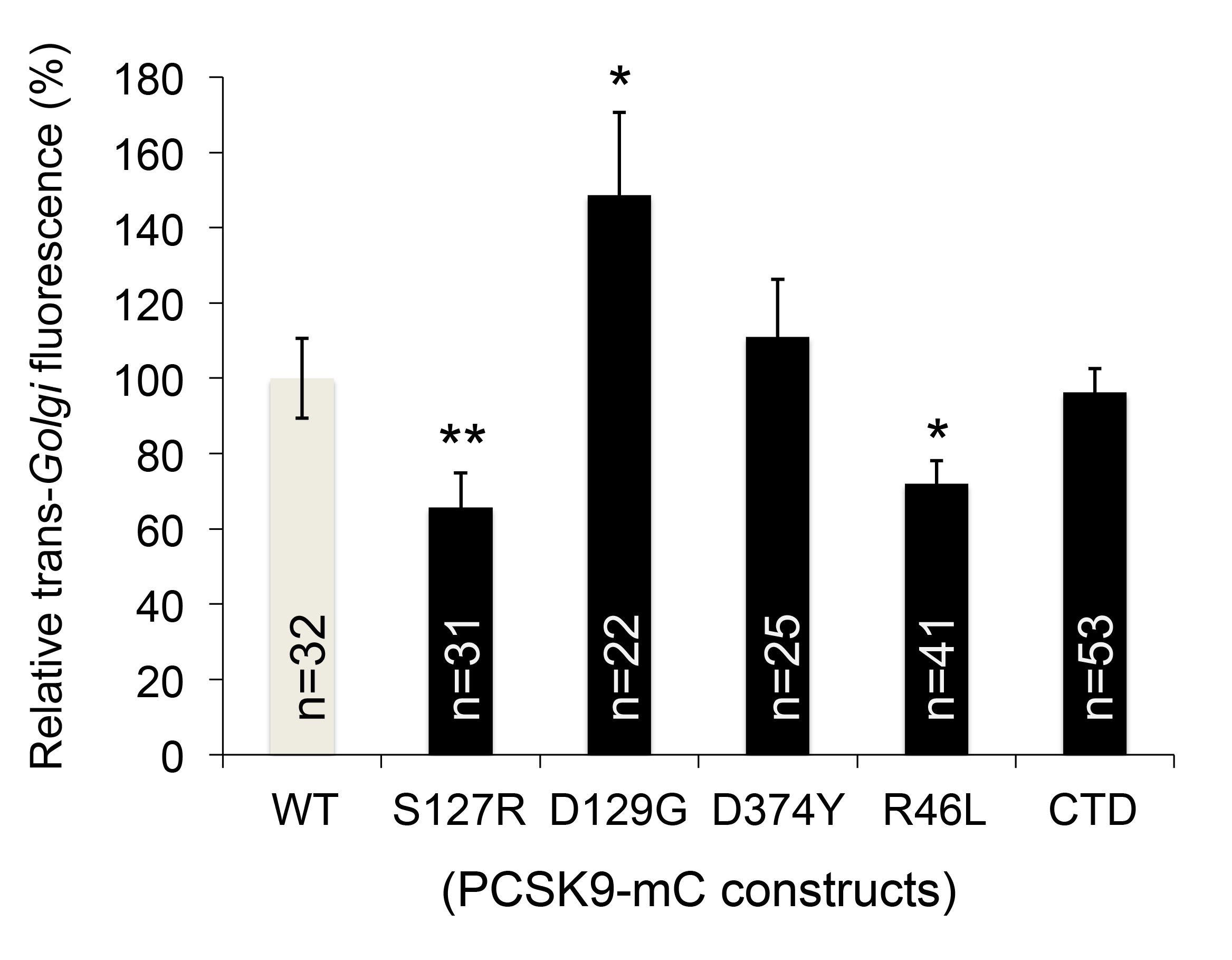

Supplement: S1 Fig — HepG2 cells were transfected with FL PCSK9-mC (n = 32), GOF S127R (n = 31), D129G (n = 22), D374Y (n = 25), LOF R46L (n = 41) or CTD alone (n = 53) and Golgi fluorescence intensities were measured as described in Material and Methods. Data are shown as the mean ± S.E.M. relative to WT PCSK9. *p ≤ 0.05, **p ≤ 0.01. (TIF) [file pone.0157230.s001.tif]
